# Ethical aspects of the application of artificial intelligence in allergology 

**DOI:** 10.5414/ALX02566E

**Published:** 2025-10-23

**Authors:** Sonja Mathes, Sebastian Seurig, Stephan Traidl, Valentina Faihs, Marta Dechant, Knut Brockow, Ulf Darsow

**Affiliations:** 1 Department of Dermatology and Allergy, School of Medicine and Health, Technische Universität München, Munich,; 2 Department of Respiratory Medicine, Allergology and Sleep Medicine, General Hospital Nuremberg, Campus North, Paracelsus Medical University, Nuremberg, and; 3 Department of Dermatology and Allergy, Hannover Medical School, Hanover, Germany

**Keywords:** AI, ethics, bioethics, allergy, trends in allergology

## Abstract

Background: Artificial intelligence (AI) offers a wide range of applications in allergology, including diagnostics and disease course prediction, consultations, real-time monitoring of allergic reactions, and support for decentralized clinical studies. Materials and methods: This review aims to highlight not only the potential applications but also the ethical aspects of using AI in allergology. Results: Initial studies demonstrate potential applications of AI in predicting provocation tests and antibiotic delabeling. However, these models from research and development have not yet been established in clinical practice, partly because ethical considerations, alongside technical challenges, and data quality issues, must be addressed. Key ethical dilemmas include bias and fairness, the principle of non-maleficence, data protection and autonomy, transparency of AI models, and questions of accountability. AI applications must be robust and reliable to prevent harm caused by erroneous recommendations. Conclusion: The use of AI in allergology requires clear guidelines based on principles such as autonomy, justice, and non-maleficence. General bioethical principles must be complemented by specific regulations for AI.

## Background 

Artificial intelligence (AI) and its capabilities have sparked broad interest, leading to a surge of publications and projects aiming to integrate these technologies into clinical practice. With such developments, ethical questions also arise in the field of allergology. However, specific ethical considerations regarding the use of AI in allergology are still rarely addressed, and there have been calls for more focused debate on this topic [2]. 

## Application of AI in allergology 

There are numerous potential applications of AI in allergology, including diagnostics, disease progression forecasting, monitoring treatment tolerability in clinical settings, real-time documentation of allergic reactions, and remote monitoring of patient data. In research, AI is used to analyze large datasets, create digital patient twins for disease modeling, and support visit planning in decentralized clinical trials [2, 3]. 

So far, only a few of these AI applications have been implemented in allergology. This is surprising given the already established use of AI in fields like dermatology, radiology, cardiology, psychiatry, and oncology [4]. For example, AI is used in dermatology for the diagnosis of malignant melanoma and has already demonstrated superior performance compared to clinicians [5]. 

Digital tools are also known in allergology, such as mobile apps for food allergy patients to find allergen-free foods [6], or decision support tools for clinicians in β-lactam antibiotic delabeling [7]. AI is now gradually entering allergology, at least in research contexts. For instance, neural networks have been used to predict outcomes of provocation tests. AI models have also been successfully applied in antibiotic delabeling and peanut allergy cases. One model for antibiotic delabeling by Moreno et al. [8] achieved strong performance, with an AUC of 0.939, sensitivity of 0.811, and specificity of 0.979. In predicting positive provocation tests for food allergies (peanut, egg, milk), neural networks have also shown promising results [9]. A model trained on data from toddlers in the LEAP cohort for predicting positive peanut challenges achieved an AUC of 0.993 [10]. While these developments indicate emerging practical use, limitations remain – beyond technical issues such as data provenance and quality, ethical concerns must also be addressed [3]. 

## Relevance of ethical questions regarding AI in allergology 

Clinical medicine and biomedical research each follow their own ethical codes, which must be considered when evaluating the use of AI [11]. For clinicians, the core tenets of the Hippocratic Oath remain applicable today; for researchers, the Declaration of Helsinki provides the guiding ethical framework [12, 13]. In medical AI, specific ethical tensions have emerged. International literature identifies transparency, reliability, fairness, and the non-harm principle as key challenges, especially in relation to patients and their privacy [11]. At present, ethical aspects of AI in allergology are only marginally addressed [2]. 

**Figure 1:**
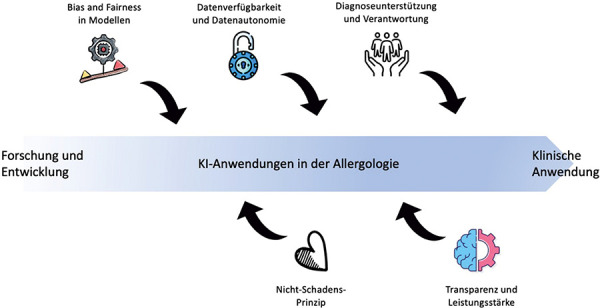
As in all areas of medicine, ethical considerations play an important role in allergology. In the context of AI applications in allergology, both in research and in clinical practice, key ethical issues include bias and fairness, the principle of non-maleficence, data availability and data autonomy, transparency and the clinical performance of AI systems, as well as questions of accountability. These areas are relevant to all stages of AI implementation in allergology, from development to clinical application.

Ethical questions require a context-specific analysis that accounts for the unique characteristics of each medical discipline [14]. This also applies to ethical considerations concerning AI. Among the numerous challenges AI introduces to various domains in allergology are those in which ethical dilemmas have already been described. This includes antibiotic delabeling [15]. Enabling such delabeling – potentially even without provocation testing – is ethically justifiable due to the opportunity costs of unnecessarily avoiding first-line antibiotics, which can contribute to antimicrobial resistance [15]. Thorough evaluation of medical indications, as well as the accurate interpretation and communication of allergy test results, have been highlighted as ethically significant. For example, patients and their families should be clearly informed about the meaning of sensitization and how it differs from manifest allergy, in order to avoid unnecessary avoidance behaviors and testing [16]. AI holds promise for use in many areas of allergy diagnostics, therapy, and monitoring [3]. However, it inevitably enters domains where ethically sensitive conflicts already exist. A comprehensive examination of relevant AI-related aspects in allergology is necessary – especially given the rapid pace of AI development – to identify sensitive ethical issues early and to explore appropriate solutions. Central ethical considerations are outlined below and illustrated in Figure 1. 

## Key ethical considerations for AI in allergology 

AI is also gaining attention in allergology [8, 10]. Its use raises questions that should account for the specific characteristics of this medical field. A comprehensive analysis of relevant aspects of AI in allergology can help identify ethically sensitive issues early and support the discussion of possible solutions. Drawing on criteria used in the international literature to evaluate AI-related ethical questions [11, 17, 18], it is possible to develop ethical frameworks tailored specifically to allergology. 

## Bias and fairness 

Access to data is essential for developing reliable algorithms. This applies to all types of AI, regardless of complexity – from logistic regression models to neural networks and large language models. To avoid bias – i.e., distortion in the data – a sound data foundation is crucial. If certain population groups are inadequately represented, AI may reinforce existing inequalities. To prevent this, the training and validation datasets used in AI must be critically assessed with regard to their provenance [3]. Various forms of bias and discrimination must be addressed, and equitable access to AI must be promoted to ensure fairness [19]. 

The FAIR principles provide a framework for distinguishing between equitable AI and systems that are more prone to bias [17]. According to these principles, AI, including allergology-related AI, should operate with findable, accessible, interoperable, and reusable data and algorithms. The fair use of AI in allergology requires equitable access to allergy care and appropriate representation of a diverse patient population. 

## Non-maleficence principle in AI for allergology 

The principle of non-maleficence – “do no harm” – is a foundational concept in bioethics, as articulated by Beauchamp and Childress [18], and historically rooted in the Hippocratic Oath. AI can cause harm when incorrect and potentially harmful recommendations are made based on flawed algorithms [20]. This is particularly critical in high-risk areas of allergology, where AI errors could endanger patient safety or even be life-threatening. 

When incorrect information is generated by AI, there is a risk that it will be passed on by clinicians and followed by patients. In allergology, misinformation – even in small details – can have serious consequences. As noted earlier, unnecessary food avoidance following poor dietary guidance for individuals with food allergies can be ethically problematic, as it introduces harm without benefit [1]. The use of AI for dietary recommendations must be approached with great caution: if unsafe foods are recommended in error, patients – especially those with primary food allergies – may face significant health risks. Models intended for use in these high-risk contexts must demonstrate excellent performance, reliability, and accuracy, particularly when used to give advise in contexts with a risk of severe allergic reactions. 

## Data availability and data autonomy 

The identifiability of individuals from data is a general concern, especially when such information may become publicly accessible against a patient’s will. For instance, patients could potentially be identified based on detailed or specific medical history data. When patient data are used to train or operate AI models, data protection, and adequate informed consent must be ensured. 

This requires, on the one hand, that patients are informed about what happens with their data, always retaining the right to choose, if their data is shared. Effective consent must be informed consent – this applies in both research and clinical contexts. On the other hand, AI models must comply with data protection standards. A particularly problematic practice is the uncritical transfer of data to third countries that, from a European perspective, lack adequate data protection guarantees. This includes, in some cases, the United States, where many major AI providers are based. 

To address this, the EU and the US have established the EU-US Data Privacy Framework, a legal agreement requiring adherence to EU data protection principles, which US companies may voluntarily adopt [21]. 

## Transparency and performance of AI 

AI can analyze vast amounts of data – including patient histories and other clinical information. This capacity enables more individualized diagnostics and treatment, moving toward precision medicine. When used appropriately, AI can help achieve optimal outcomes for patients. In allergology, where medical histories and sensitization profiles vary greatly between individuals, a case-by-case assessment is often necessary. Models that effectively handle complex clinical scenarios must be capable of processing and retrieving a large volume of case-specific and contextual data. 

Unsupervised models such as neural networks have proven particularly powerful. However, they are often referred to as “black-box” systems because their internal processes are not transparent. There is an inverse relationship between a model’s performance and the interpretability of its results. Approaches like explainable AI aim to address this problem by making AI decisions more comprehensible [3, 5, 10]. 

## Decision support and responsibility 

The influence of AI on human decision-making is sometimes viewed critically. Especially when AI systems are highly effective and easy to use, there is a temptation for users to rely heavily on their outputs. AI has since evolved into tools known as Clinical Decision Support Systems (CDSS). These are already used in several medical specialties – such as dermatology for image recognition – and in some cases, they outperform clinicians, for example in the detection of malignant melanomas [4, 5]. 

As tools for decision support, AI applications have the potential to enhance patient well-being – which, according to the principle *salus aegroti suprema lex*, should be the foremost goal of clinical care. However, this potential benefit must be weighed against the risk of automation bias: the uncritical acceptance of AI-generated suggestions. Alongside ethical concerns about moral responsibility for errors, legal liability issues also arise. There is a prevailing tendency to place ultimate responsibility on the human user who chose to rely on the AI system. 

This line of reasoning tends to hide out the “black-box” nature of many current AI systems, which are not easily interpretable and leave users unable to fully understand how decisions are made – yet still require them to act on those outputs. Legal discourse in this area is ongoing and far from settled [22]. 

## Discussion 

Specific features of allergology – such as the diagnosis of vulnerable populations like children, or the risks associated with provocation testing – give rise to ethical considerations that intersect with key domains such as bias and fairness, the non-maleficence principle, data availability and autonomy, transparency and performance of AI, and responsibility in clinical decision-making. 

### Ethical challenges of AI in allergology research 

The ethical use of AI in research is governed by different regulations than its application in clinical practice. Nevertheless, ethical safeguards and institutional frameworks are also in place to support responsible research. Ethical approval is a standard requirement for research projects involving human participants or identifiable human biological materials. Physicians have an obligation to seek ethical consultation for such studies. In Germany, these studies must be formally submitted for review, and a medical ethics committee must be asked for an ethics vote [12, 13]. 

Beyond professional regulations, the use of AI must comply with additional legal frameworks, including the recently adopted EU AI Act, the Medical Device Regulation, and the General Data Protection Regulation (GDPR). The primary responsibility for ensuring compliance with both legal and ethical standards lies with allergologists [22]. 

Interdisciplinary discussions that incorporate ethical perspectives can help equip clinicians and researchers with the necessary arguments to use AI responsibly and in the best interests of their patients in daily practice. 

### Ethical challenges of AI in allergology practice 

In individual clinical cases, arguments from general ethical discourse can be applied. Currently, only limited literature addresses ethical questions specifically related to AI in allergology [2, 15, 16], which has led to recent calls for greater attention to these issues [2]. Due to the scarcity of publications on this topic, it is necessary to rely on established principles from bioethics and research ethics. 

Existing ethical frameworks offer valuable orientation, although they may not directly address the specific needs of allergology. These include the significant heterogeneity of clinical presentations and the importance of data protection in managing sensitive allergological patient data. Foundational works in ethics, such as the bioethical principles by Beauchamp and Childress, provide the framework for assessing allergology-specific ethical questions. 

The principles of autonomy, non-maleficence, beneficence, and justice – repeatedly emphasized in this article – apply to all patients, including those in allergology [18]. Resolving conflicts between these principles in individual cases and acting in the patient’s best interest is the responsibility of the treating clinicians. 

Although ethical codes continue to evolve, they do not yet adequately address AI-specific concerns. For example, the 2024 revision of the Declaration of Helsinki includes no explicit reference to AI and related ethical issues [23]. The responsibility for ethical reflection in developing and applying AI in allergology ultimately lies with allergologists themselves. As AI becomes increasingly integrated into medical practice, further discussion will be necessary – particularly when these technologies are applied to human subjects or biological materials – and concrete strategies must be developed. 

### Approaches to address ethical challenges in AI for allergology 

Strategies for addressing ethical issues related to AI in allergology can be found in established methods of applied ethics, such as moral case deliberation, particularly in clinical practice. This method supports the structured analysis of ethically complex cases. It follows a seven-step, moderated process to move from a moral conflict in patient care to an individualized decision on how to resolve the issue. 

All individuals involved in the case participate in this process, including clinicians, nursing staff, and ethicists. The final resolution incorporates the specific circumstances of the case, as well as the moral values and expectations of all parties. 

Criticism of this method includes its time-intensive nature, the personnel required, and the limited availability of qualified ethicists. Nevertheless, moral case deliberation has advantages: it promotes shared decision-making, can reduce moral distress, and strengthens the ethical competence of participants. Its structured process ensures that all voices are heard and encourages openness to arguments from all perspectives — contributing to a thorough understanding of the case [14]. 

## Summary and outlook 

AI could be of great value for research, diagnostics, and therapy in allergology in the future. However, the ethical considerations that must be addressed are complex — particularly with regard to bias, fairness, the principle of non-maleficence, data protection, and the responsibility of clinicians. 

Ethical use of AI in allergology requires clear guidelines and interdisciplinary discussions to address specific challenges and ensure adherence to fundamental bioethical principles. 

## Authors’ contributions 

SM conceptualized the study, drafted the manuscript, and prepared the figure for visualization. SS contributed to drafting the manuscript and participated in reviewing and editing. ST, MD, VF, and KB reviewed and edited the manuscript. UD supervised the writing process and also contributed to reviewing and editing the manuscript. 

## Funding 

This project received no external funding. 

## Conflict of interest 

The authors have no conflict of interest to declare.
